# Comparison of Monetary Policy Actions and Central Bank Communication on Tackling Asset Price Bubbles—Evidence from China’s Stock Market

**DOI:** 10.1371/journal.pone.0166526

**Published:** 2016-11-16

**Authors:** Ou Sun, Zhixin Liu

**Affiliations:** School of Economics and Management, Beihang University, Beijing, China; Universidad Veracruzana, MEXICO

## Abstract

We examine the different effects of monetary policy actions and central bank communication on China’s stock market bubbles with a Time-varying Parameter SVAR model. We find that with negative responses of fundamental component and positive responses of bubble component of asset prices, contractionary monetary policy induces the observed stock prices to rise during periods of large bubbles. By contrast, central bank communication acts on the market through expectation guidance and has more significant effects on stock prices in the long run, which implies that central bank communication be used as an effective long-term instrument for the central bank’s policymaking.

## Introduction

The 2008 financial crisis and the high volatility of China’s stock market in recent years highlight the importance of financial stability. There has been a hot debate on whether asset price bubbles have wider economic consequences and whether monetary policy should react to it [1]. Research of Liu et al. [2] proves that as an emerging market, China’s stock market has larger volatility than stock market of the US (a mature market). However, there have been limited studies on how different kinds of monetary policy instruments should react to it. In recent years, the emergence of unconventional monetary policy has caused widespread concern among academia and industry, bringing the comparison of conventional and unconventional monetary policy on the table. Previous studies mention that transmission efficiency of conventional monetary policy is lower in developing than in developed countries because of the financial market incompleteness. Whereas central bank communication, one of the unconventional monetary policies, can enhance the predictability of monetary policy and guide the market effectively by expectation management, and play a central role in macroeconomy supervision [3]. In this paper, we try to tackle the problems of whether monetary policy should react to asset price bubbles in China and what instrument is more efficient in doing this by analyzing different effects of conventional and unconventional monetary policy on asset prices using data of China’s stock market, a developing market with unconventional monetary policy used frequently in the past two decades.

In the first place, there are two problems we have to tackle—identification of the bubble, and the endogeneity problem. Previous studies usually identify the bubble by extracting the fundamental component from the asset prices, but the frequently used discounted cash flow method of calculating the fundamental component is not suitable to China’s stock market with few dividends. So we adopt the residual income valuation model developed by Feltham and Ohlson [4](*F-O* model) in this paper. Further, we construct a Time-varying Parameter Structural Vector Autoregression (TVP-SVAR) model of three variables, the monetary policy indicator, the observed asset prices and the fundamental component of asset prices, to identify the bubble by distinguishing different impacts of monetary policy on the observed asset prices and the fundamental component.

The endogeneity problem means that monetary policy and the asset price bubble not only influence each other, but also both can be affected by many other factors. Previous studies have used different methods to overcome the endogeneity problem, such as the use of high-frequency data [5–6], the identification technique based on heteroskedasticity [7–9], instrument variable method and event study. In this paper, we set monetary policy shocks to be exogenous in the TVP-SVAR model to overcome the endogeneity problem.

We use quarterly data in this paper. Therefore, when constructing the communication indicator, it is necessary to give weights to communication events in different months in a quarter because of their timeliness. To be specific, the effect of central bank communication is quite timely because of its high frequency. Whereas the accumulation and bursting of a bubble are relatively longer processes. Reconciling the two of them is another issue needed to be addressed. To do this, we give weights to communication events chronologically to differentiate the orders and effects of them by the method of variance decomposition. There are some previous studies on central bank communication putting weights on different code words according to their intensity [5–7]. To the best of our knowledge, using weights on the timing of a communication event is the first try in this field.

This paper has three contributions. First, it is the first time to compare the different effects of monetary policy actions and central bank communication on asset price bubbles in China, which is not only a comparison of direct and indirect, but also of conventional and unconventional monetary policy instruments. Second, taking account of the timeliness of central bank communication, we give weights to communication events chronologically to differentiate the orders and effects of them, which makes the communication indicator more applicable. Third, we use the *F-O* model based on residual income to calculate the fundamental component of stock prices, which is more suitable for China’s stock market with few dividends.

Through impulse response analysis of the TVP-SVAR model, we find that there are positive responses of the observed asset prices to contractionary monetary policy actions during periods of high bubbles. This is based on the fact that the fundamental component of asset prices has negative responses to monetary policy shocks whereas the bubble component has positive responses. Different from these results of monetary policy actions, central bank communication has more significant long-term effects on asset prices, mainly through its role of expectation management.

## Literature Survey

### Monetary Policy Actions

Whether monetary policy should take account of asset prices is one of the most discussed policy issues in recent years. Akram et al. [5], Christiano et al. [6], Borio [7] argue that there will be financial instability once the bubble bursts and monetary policy should react to asset price fluctuations to prevent the accumulation of bubbles. From the other point of view, Bernanke and Gertler [8,9] and Rigobon and Sack [7] argue that monetary policy should react to asset price movements only to the extent that they affect expected inflation. Galí [10], Galí and Gambetti [11] prove that an increase of the interest rate in response to a growing bubble will enhance the fluctuations in asset prices when bubble’s proportion is larger in the asset price.

### Central Bank Communication

Communication was used widely and frequently during the 2007–2008 financial crisis by central banks as an important unconventional monetary policy instrument, which leads to a hot discussion about its function of expectation management and market regulation. Blinder et al. [12] point out that central bank communication can be used to manage expectations by ‘creating news’ and ‘reducing noise’. Most of the subsequent studies give support to their work except for Friedman [13], who compares the two forms of unconventional monetary policy and concludes that central bank communication has been less successful than large-scale asset purchases, the other widely used unconventional monetary policy.

Researches on central bank communication mainly focus on the impacts of communication about monetary policy stance, macroeconomic outlook and financial stability on the yield curve [14], stock prices and bond yields [15,16], exchange rates [17], and expectation [18]. As to the form of communication, some focus on oral communication [19], some focus on written statements [20] and others consider both two forms [16].

### Comparison of Monetary Policy Actions and Central Bank Communication

The mechanisms of monetary policy actions and central bank communication are different. Previous studies generally believe that communication has more significant effects on the long-term yields by influencing the market expectations [21, 22]. Kliesen and Schmid [23] study the different effects of them on inflation expectation and conclude that Federal Reserve communication reduces uncertainty about the future rate of inflation, whereas surprises in monetary policy actions increase uncertainty about the path of the inflation rate. Comparison of the two instruments in China’s case involves the effect of monetary policy on inflation expectation [24] and macroeconomic situations [20, 25].

### Link of Monetary Policy Actions and Central Bank Communication

As two monetary policy instruments, monetary policy actions and central bank communication have certain connections. Central bank communication is considered to be another way of monetary policy transmission before the actual monetary policy actions, which is called the ‘market-expectations channel’ of monetary policy transmission by Neuenkirch [26] and Gertler and Karadi [27]. Through this channel, communication enhances the predictability of monetary policy, shortens the transmission lag and improves the efficiency of monetary policy actions [28].

Some studies explore further the conditions on which central bank communication can function better. Brand et al. [22] and Jansen and De Haan [29] argue that communication is effective only on the condition that it is consistent with monetary policy actions.

To conclude, there are limited researches studying the differences of the two monetary policy instruments in affecting stock prices and controlling bubbles, especially in the background of China’s undeveloped stock market with higher fluctuations. In this paper, we use data of China’s stock market and monetary policy to give a first try in this field.

## Method and Data

### Method

The methodology of this paper is based on the theory of rational bubbles raised by Blanchard and Watson [30] and the analysis of Galí and Gambetti [11]. The theory of rational bubbles suggests that asset prices can be decomposed into a bubble component and a fundamental component, and with the hypothesis of investor’s rational expectations and rational actions, there could exist asset price bubbles even under the condition of no arbitrage. Investors would buy the assets undervalued, expecting to sell them at a higher price, thus push up the asset price gradually. According to this theory, the fundamental component responds negatively to monetary policy shocks, whereas the bubble component grows at the rate of the interest rate. As analyzed by Galí and Gambetti [11], we can figure out the existence of bubbles when the observed asset prices rise in response to an interest rate increase. As to central bank communication, a similar analysis can be applied.

Based on the analysis above, we build the following three-variable Time-varying Parameter Structural Vector Autoregression (TVP-SVAR) model (1) with two lags (determined based on the highest marginal likelihood), in which we consider time-varying of both the parameters and the variance covariance matrix of the model (stochastic volatility) as in Primiceri [31].

$$Y_{t}=a_{t}+B_{t,1}Y_{t-1}+B_{t,2}Y_{t-2}+\varepsilon_{t}$$ (1)

Where *Y*_*t*_ *=* [*f*_*t*_, *m*_*t*_, *p*_*t*_]*'* is a 3×1 vector of the observed variables at time *t*. *f*_*t*_ is the fundamental component of asset prices. *m*_*t*_ represents monetary policy indicator, and *p*_*t*_ is the observed asset prices. *a*_*t*_ is a 3-dimensional column vector of intercepts. *B*_*t*_,_*p*_ (*p* = 1, 2) is a 3×3 matrix of the autoregressive coefficients. Residual *ε*_*t*_ follows the distribution *N*(0, Ω_*t*_), where Ω_*t*_ is the variance covariance matrix. The subscript *t* of *a*_*t*_, *B*_*t*_,_*p*_(*p* = 1, 2) and Ω_*t*_ shows that they are all time-varying, and Ω_*t*_ indicates the stochastic volatility of the model.

Following Primiceri [31], we assume that the VAR coefficients follow the random walk: *β*_*t*_ = *β*_*t*−1_ + *μ*_*β*,*t*_, where *β*_*t*_ *=* [*vec*(*a*_*t*_)*'*, *vec*(*B*_*t*,*1*_)*'*, *vec*(*B*_*t*,*2*_)*'*]*'*.

We define the covariance matrix $\Omega_{t}=A_{t}^{-1}H_{t}(A_{t}^{-1}{)}^{\prime }$. Where *A*_*t*_ is a lower triangular matrix with diagonal elements of 1, *H*_*t*_ is a diagonal matrix.

$$A_{t}=\left[\begin{array}{ccc} 1 & 0 & 0\\ \alpha_{21,t} & 1 & 0\\ \alpha_{31,t} & \alpha_{32,t} & 1 \end{array}\right];\;H_{t}=\left[\begin{array}{ccc} h_{1,t} & 0 & 0\\ 0 & h_{2,t} & 0\\ 0 & 0 & h_{3,t} \end{array}\right]$$

We assume the vector of non-zero and non-one elements of matrix *A*_*t*_ (stacked by rows) evolves as random walk: *α*_*t*_ = *α*_*t*−1_ + *μ*_*α*,*t*_. Let *h*_*t*_ be the vector of the diagonal elements of *H*_*t*_ and we assume it evolves as geometric random walk: In *h*_*t*_ = ln *h*_*t*−1_ + *μ*_*h*,*t*_. Further, all the innovations in the model, *ε*_*t*_, *μ*_*β*,*t*_, *μ*_*α*_,_*t*_, and *μ*_*h*_,_*t*_ are assumed to be mutually uncorrelated at all leads and lags and follow the jointly normally distribution below.

$$\left[\begin{array}{c} \varepsilon_{t}\\ \mu_{\beta ,t}\\ \mu_{\alpha ,t}\\ \mu_{h,t} \end{array}\right]\sim N\left[0,\left[\begin{array}{cccc} I_{3} & 0 & 0 & 0\\ 0 & \Sigma_{\beta } & 0 & 0\\ 0 & 0 & \Sigma_{\alpha } & 0\\ 0 & 0 & 0 & \Sigma_{h} \end{array}\right]\right]$$

Where *I*_3_ is a 3-dimensional identity matrix, Σ_*β*_, Σ_*α*_, and Σ_*h*_ are positive definite matrices.

To overcome the endogeneity problem, we set monetary policy shocks as exogenous to the observed asset prices, which means that monetary policy indicator doesn’t respond contemporaneously to shocks of the observed asset prices. To be specific, we set the simultaneous response coefficients of monetary policy indicator to asset price indicator in the coefficient matrix (covariance matrix) to be 0 in the TVP-SVAR model.

The estimated coefficients are multiplied by allowing random variation of the parameters. So we refer to Primiceri [31] to apply the Markov Chain Monte Carlo (MCMC) methods in the context of a Bayesian inference to estimate the model, in which Gibbs sampling procedure is used to obtain the joint posterior distribution of the parameters in the model. We assume that the initial states for *β*_*t*_, *α*_*t*_, ln *h*_*t*_, and the hyper parameters Σ_*β*_, Σ_*α*_, and Σ_*h*_ are independent of each other. The priors for the hyper parameters Σ_*β*_, Σ_*α*_, and Σ_*h*_, are assumed to be distributed as independent inverse-Wishart. The priors for the initial states of *β*_*t*_, *α*_*t*_, and ln *h*_*t*_ are assumed to be normally distributed.

### Discussion about the Exogenous Assumption

**Fig 1:**
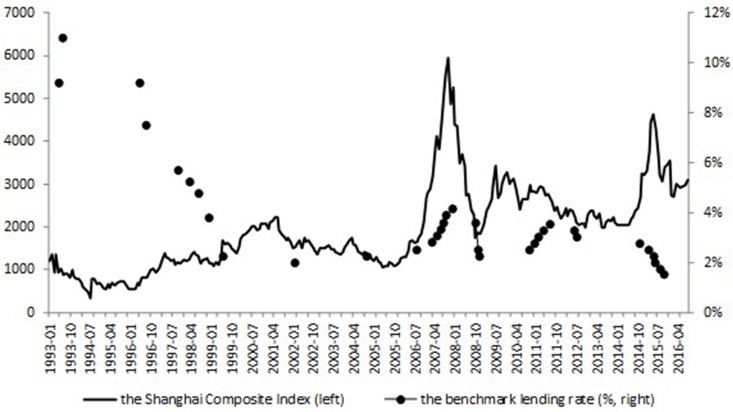
The Shanghai Composite Index and the benchmark lending rate. This figure plots the Shanghai Composite Index (left scale) and the benchmark lending rate (right scale, %). Source: Shanghai Stock Exchange (SSE), and website of the PBOC.

**Fig 2:**
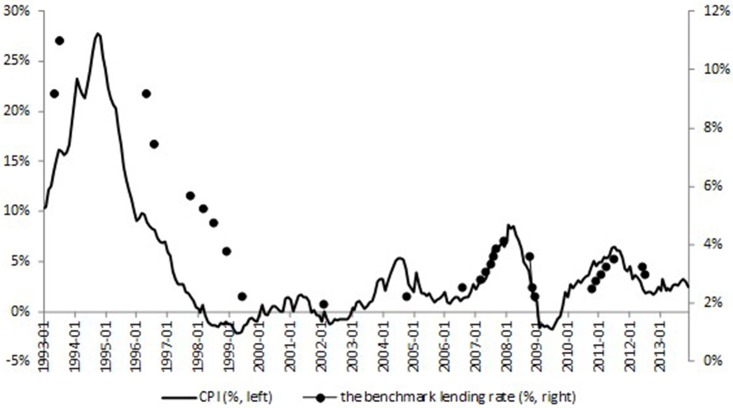
The CPI and the benchmark lending rate. This figure plots the CPI (left scale, %) and the benchmark lending rate (right scale, %). Source: *Wind* financial database, and website of the PBOC.

**Fig 3:**
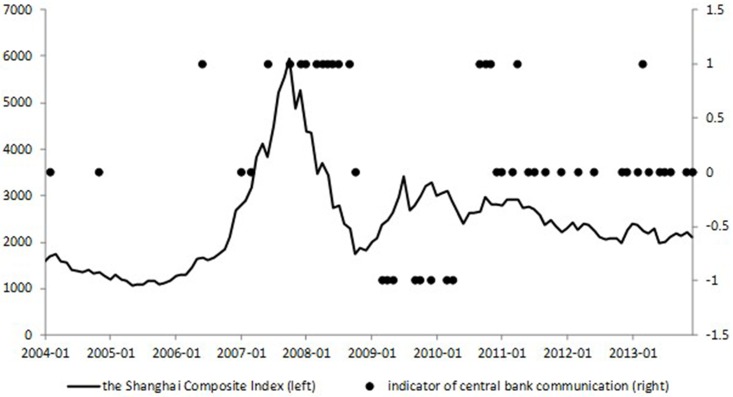
The Shanghai Composite Index and central bank communication indicator. This figure plots the Shanghai Composite Index (left scale) and central bank communication indicator (right scale). Source: Shanghai Stock Exchange (SSE), and own calculations.

**Fig 4:**
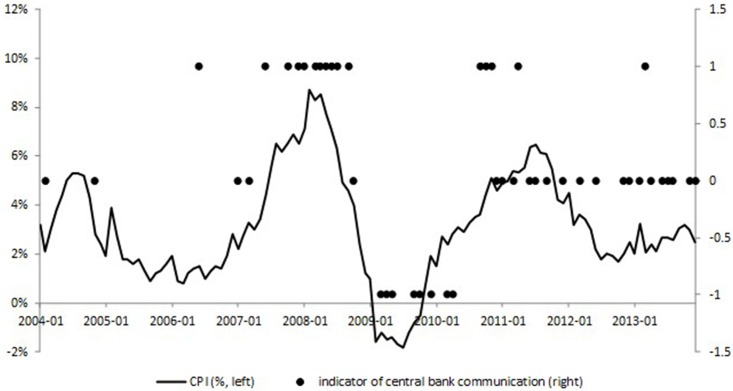
The CPI and central bank communication indicator. This figure plots the CPI (left scale, %) and central bank communication indicator (right scale). Source: *Wind* financial database, and own calculations.

The exogenous assumption of monetary policy shocks to the observed asset prices is the key of our model. In this part, we give a discussion about the rationality of its application in China. For the central bank of China (the People's Bank of China, the PBOC), there are mainly four ultimate objectives, economic growth, price stability, full employment and balance of payments. Inflation is the key factor determining monetary policy decisions, whereas financial stability is just one of the factors it accounts for, which has been communicated by authorities of the PBOC in public for several times. Only when the financial situation affects or contains enough information of the trend of inflation, can it affect policy decisions. We try to justify this assumption and explain it through Figs 1–4.

Figs 1 and 2 show trend of the benchmark lending rate and its relationship with the Shanghai Composite Index and the CPI (detailed descriptions of the data used in this paper will be stated in Section “Data”). We can see from the figures that interest rate adjustments mainly follow the trend of the CPI, and there is no apparent causal link between interest rate adjustments and the stock market index. To be more specific, we give detailed explanations of the three periods with interest rate adjustments of high-frequencies, 1993–1994, 2007–2008, 2010–2011.

During the year of 1993, the PBOC raised the interest rate considering the hyper-inflation problem during that time, even though the stock market index is hovering around the low point of 1000. The CPI went down since late 1994, after when the interest rate was also turned down gradually. Not until then did the stock market realize a recovery.

Period 2007–2008 is also a clear example. It is almost seven months between the two interest rate rises on August, 19, 2006 and March, 18, 2007, during which time the stock market index rose from 1598.02 to 2930.48 for about 83%. We can see that even with the apparent bubble accumulating, the stock market instability has not become the causation of the PBOC to raise the benchmark interest rate. It is only when there appears instability in the inflation, the CPI, which is 2.7% in February, 2007 rising from 2.2% of January, 2007, that the monetary policy decision changed, followed by another five interest rate rises. We can further confirm the exogeneity of monetary policy to the stock market by the fact that the stock market index turned down since October, 2007 from the top of 6092.06, however there was another interest rate rise after that on December, 21, 2007, considering that the CPI is still high in November, 2007 at 6.9%.

Similar situation happened during middle of 2010 and middle of 2011. There were a series of interest rate rises when the CPI rose up whereas there were no apparent bubbles in the stock market.

To conclude, monetary policy does not react to the stock market in China, which makes the exogenous assumption applicable in this paper, even with quarterly data.

When it comes to central bank communication, we can get the same conclusion. Figs 3 and 4 show regularity of central bank communication, in which +1, 0, -1 represent tightening, neutral, and easing monetary policy inclinations, respectively (We give detailed introduction of the measurement of central bank communication indicator in Section “Measurement of Central Bank Communication”). The exogeneity of central bank communication can be concluded through the similar logic to the above analysis. The neutral communications were applied to stabilize the market sentiment and were not inclined. Apart from these samples, the tightening and easing communications during October, 2007 and October, 2008, March, 2009 and April, 2010, and September, 2010 and April, 2011 indicated apparently that they all happened along with the high fluctuations (climbing up or going down) of the CPI, not the stock market index.

Therefore, we can justify our assumption that monetary policy, not only conventional, but also unconventional monetary policy, reacts to the inflation index and is exogenous to the situation of the stock market.

### Data

For the three variables in the TVP-SVAR model, we use the logarithmic form of the benchmark lending rate log(*r*_*t*_) as the indicator of monetary policy actions and the communication index *c*_*t*_ as the indicator of central bank communication. Also, we give the results of the overnight interbank lending rate as the monetary policy actions indicator for robustness test. The logarithmic difference form of the Shanghai Composite Index Δ log(*Index*_*t*_) is applied as the observed asset prices and the logarithmic form of the fundamental component log(*funda*_*t*_) is obtained by calculation. We exhibit detailed descriptions of monetary policy indicator selection, constructions of central bank communication index and the fundamental of stock prices below.

Our sample period is 1994Q1-2013Q4 for the estimation of monetary policy actions, 2003Q1-2013Q4 for the estimation of central bank communication, considering the data availability. We also give results of monetary policy actions during period of 2003Q1-2013Q4 in Section “Empirical Results of Central Bank Communication”, to make the results comparable. The sample contains the several ups and downs of China’s stock market in recent years, including the accumulation, expansion, and bursting of bubbles, thus can be an appropriate representative.

Data resources of this paper are as follows. We get the benchmark lending rate from the website of the PBOC, the Shanghai Composite Index, net assets and return of equity of the stock market (to calculate the fundamental component) from the *Wind* financial database.

### Measurement of Monetary Policy Actions

We select the benchmark lending rate as the monetary policy indicator. There are two reasons why we think of it as appropriate. First, the intermediate target of China’s monetary policy has transformed gradually from quantitative indicators to price-based indicators, and the lending rate can fully represent monetary authorities’ intentions. Second, the applicability of lending rate as monetary policy indicator in China has been affirmed by the work of Feng and He [32] and Ouyang [20] on monetary policy transmission effects.

We also use the more market-oriented monetary policy indicator, the overnight interbank lending rate, to verify the robustness of our results.

### Measurement of Central Bank Communication

There are mainly two forms of the PBOC communication. One is written communication, thus reports regularly published by the Monetary Policy Committee (MPC). The other form is oral communication, including press conferences of the PBOC, statements, speeches, and interviews made by members in the MPC. We get written communication events through the official website of the PBOC and the oral communication events through the Internet by keyword searching. When searching the keyword, we only focus on oral communications of the governor of the PBOC, Mr. Zhou Xiaochuan, and nine successive deputy governors, whose tenures should be accounted for to make sure the communication events we obtain happened within their tenures. The search commands we use are the name of the governors together with the term ‘monetary policy’ to extract all relevant events, and we only select each event once on the basis of its first recording to avoid repeat counting. The oral communication events are summarized in Table 1. Summary and classification of both oral and written communication are shown in Table 2.

**Table 1:** Summary of Tenure and Oral Communications of Governors of the PBOC.

	Tenure	Communication Events			
		Neutral	Tightening	Easing	Total
**Governor**					
Zhou Xiaochuan	2002.12-	38	26	11	75
**Deputy Governor**					
Yi Gang	2007.12-	12	10	6	28
Pan Gongsheng	2012.6-	1	0	0	1
Li Dongrong	2012.7-	1	0	0	1
Hu Xiaolian	2005.8–2015.2	10	2	3	15
Wu Xiaoling	2000.2–2007.12	11	10	5	26
Su Ning	2003.11–2010.6	8	3	12	23
Xiang Junbo	2004.7–2007.7	1	0	0	1
Ma Delun	2008.1–2011.11	1	3	4	8
Liu Shiyu	2006.6–2014.11	0	0	1	1
Total		83	54	42	179

This table reports summary of oral communication events of the governor of the PBOC, Mr. Zhou Xiaochuan, and nine successive deputy governors.

**Table 2:** Summary of All the Communication Events.

Form	Neutral	Tightening	Easing	Total
Written Communication	60	10	18	88
Oral Communication	83	54	42	179
Total	143	64	60	267

This table reports number of all communication events in our sample, including written and oral communication.

The construction of central bank communication indicator is a key to our estimation. There are generally two methods to build this indicator, ‘content analysis’ [15, 33] and ‘narrative approach’ [34–36], both define a dummy variable according to the event’s information content. For example, Ehrmann and Fratzscher [33] define +1 as tightening monetary policy, 0 as neutral inclination, and -1 as easing inclination. Narrative approach is different from content analysis that it pays more attention to the transformation point of the monetary policy stance.

We define communication events following Ehrmann and Fratzscher [33] in this paper. To keep in line with the work in ‘content analysis’, we also try to reduce the chance of misclassification of the statements by having two persons analyze all the communication events independently.

We use quarterly data in this paper, so the sequence of the communication event is an important factor in the construction of the quarterly communication indicator. Effects of events happen in the first month in a quarter are definitely different from those of the third month, and it would be biased to just add assignment of each communication event in a quarter to get the quarterly indicator. Therefore, we use the method of variance decomposition to determine the weights that should be put on the communication events happening in the first, second, and third month in each quarter to reflect this timeliness.

We set a VAR model with two lags (derived by AIC) in the following form.

$$y_{t}=\beta_{1}y_{t-1}+\beta_{2}y_{t-2}+\varepsilon_{t}$$ (2)

Where *Y*_*t*_ = [*ir*_*t*_, *c*_*t*,*1*_, *c*_*t*,*2*_, *c*_*t*,*3*_] is a vector of the short-term interest rate *ir*_*t*_——the overnight interbank lending rate——and communication indicators of the three months in a quarter, *c*_*t*,*1*_, *c*_*t*,*2*_, and *c*_*t*,*3*_, which are calculated by adding the assignments of the events within each month. Through the establishment of the VAR model, we get results of the variance decomposition, in which the contributions of the shock of *c*_*t*,*1*_, *c*_*t*,*2*_, and *c*_*t*,*3*_ to the fluctuation of *ir*_*t*_ are the weights that should be put on the communication events happening in the first, second, and third month. The results we get are 0.4299, 0.0825 and 0.4876, respectively. Therefore, the final communication indicator is constructed as the weighted sum of the three month indicators.

$$c_{t}=0.4299c_{t,1}+0.0825c_{t,2}+0.4876c_{t,3}$$ (3)

There are some other studies giving weights to variables according to the sequences that we can refer to. For example, Del Negro et al. [37] study the relationship between forward guidance and the federal funds rate, in which they provide different penalties on deviations of the interest rate path over the short, intermediate, and long horizons to explain the forward guidance puzzle.

### Calculation of the Fundamental Component of Stock Prices

We use data of stocks in the Shanghai Stock Exchange (SSE) to calculate the fundamental component of asset prices, in consistent with the use of the Shanghai Composite Index in our model. We apply the residual income valuation model developed by Feltham and Ohlson [4](*F-O* model) to calculate the fundamental of stock prices [38, 39].

$$V_{t}=BV_{t}+\overset{N}{\underset{i=1}{\sum }}\frac{(ROE-\rho )\times BV_{t+i-1}}{{\left(1+\rho \right)}^{i}}$$ (4)

Where *V*_*t*_ is the price of the fundamental at time *t*. *BV*_*t*_ is the net worth at time *t*, which we use the averaged net assets per share of SSE. *N* is the number of periods investors can predict on the stock market, representing investors’ prediction ability. We assume *N* to be 10 years. *ROE* is the return on equity, which we apply the average return on equity of the previous 10 years of SSE. *ρ* is the risk-free rate, which we adopt data of the 10-year Treasury bond yield. The data source is *Wind* financial database.

**Fig 5:**
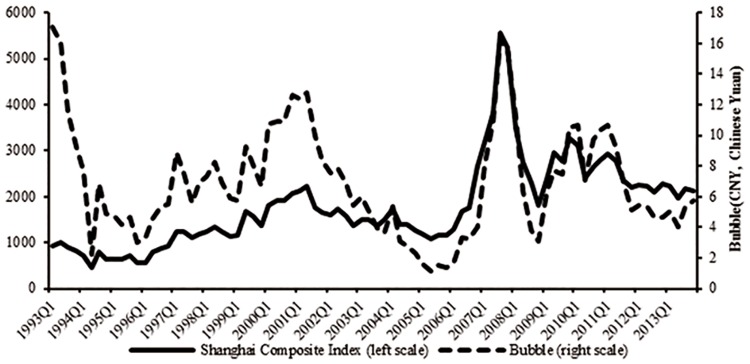
Shanghai Composite Index and bubble. This figure plots the Shanghai Composite Index (left scale) and the bubble size (CNY, Chinese Yuan). Source: Shanghai Stock Exchange (SSE), and own calculations.

**Fig 6:**
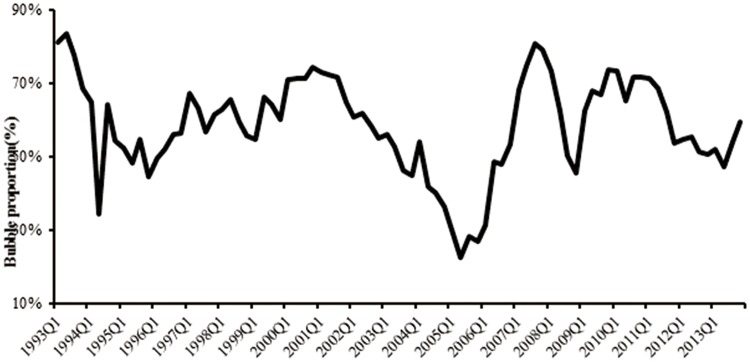
Proportion of the bubble component. Source: Own calculations.

We calculate the fundamental prices during period of 1993–2013 and find our results quite consistent with those obtained by Chen et al. [40] using the dynamic residual income valuation model, demonstrating the applicability of *F-O* model and the accuracy of our calculation. Further, we get the approximate bubble size by excluding the fundamental component from the average stock price of SSE (data source: *Wind* financial database). Fig 5 plots the Shanghai Composite Index and the bubble, from which we can see that the bubble moves closely with the stock index and can be a good fit for the several ups and downs of China’s stock market. Fig 6 shows the approximately calculated bubble component proportion in asset prices. We can see that the percentage of the bubble component is over 60% even in the bear market, which indicates the excessive bubble problem in China’s stock market.

## Empirical Results of Monetary Policy Actions

To compute the posterior estimates of the parameters, we apply the Gibbs sampling procedure by drawing *M* = 10,000 samples after the initial 1,000 samples are discarded, which is determined by the convergence diagnosis (CD test) results shown in Table 3. We estimate the TVP-SVAR model for different lag lengths and the optimal lag number is 2 based on the highest marginal likelihood.

**Table 3:** Coefficient Results of TVP-SVAR Model, monetary policy actions.

Parameter	Mean	StDev	95%U	95%L	Geweke	Inef.
(Σ_*β*_)_1_	0.23	0.03	0.18	0.29	0.001	5.23
(Σ_*β*_)_2_	0.23	0.03	0.18	0.29	0.495	4.71
(Σ_*α*_)_1_	0.64	0.26	0.35	1.35	0	46.53
(Σ_*α*_)_2_	0.4	0.07	0.28	0.57	0.68	10.31
(Σ_*h*_)_1_	0.56	0.17	0.34	1.02	0.103	36.55
(Σ_*h*_)_2_	0.55	0.16	0.34	0.93	0.565	23.09

This table reports the coefficient results of the model: the posterior mean, the standard deviation, critical values of the 95% confidence interval, the convergence diagnosis results of Geweke [41], and the inefficiency factor of the parameters. The estimates are multiplied by 100.

**Fig 7:**
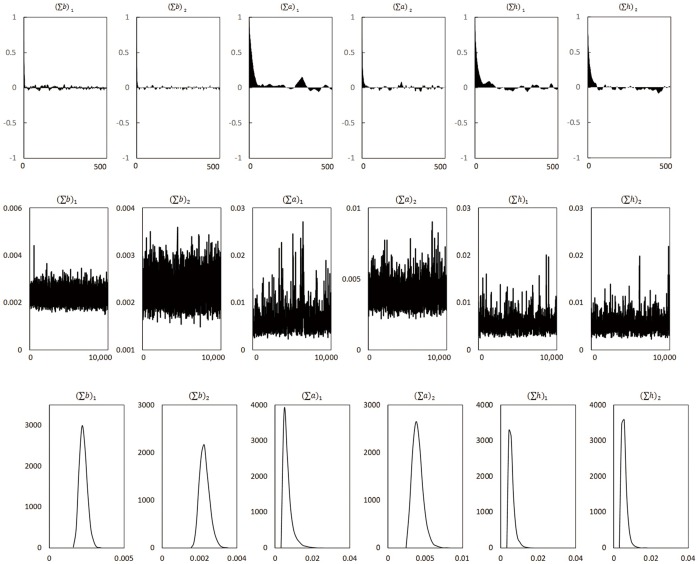
Coefficient results of TVP-SVAR model, monetary policy actions. This figure shows the autocorrelation equation of the parameters (the first row), the sample path of the parameters (the second row), and the posterior density of the parameters (the third row) in the TVP-SVAR model with monetary policy indicator the benchmark lending rate.

Table 3 and Fig 7 are the main results of the key parameters in the model. Table 3 presents the posterior mean, the standard deviation, critical values of the 95% confidence interval, the convergence diagnosis results of Geweke [41], and the inefficiency factor of the parameters, respectively. The null hypothesis of CD test is the convergence of the posterior distribution, and it cannot be rejected proved by the results here. The inefficiency factor is quite low, indicating the efficient sampling of the parameters and state variables, on the basis that the more the unrelated samples (number of sampling/inefficiency factor), the higher the sampling efficiency. Fig 7 shows the autocorrelation equation, the sample path and the posterior density of the parameters. We can see from the correlation diagram that the correlation drops gradually and tends to be stable, which means the sampling method applied can obtain some relatively low correlated samples. The sample paths also have stable trends.

**Fig 8:**
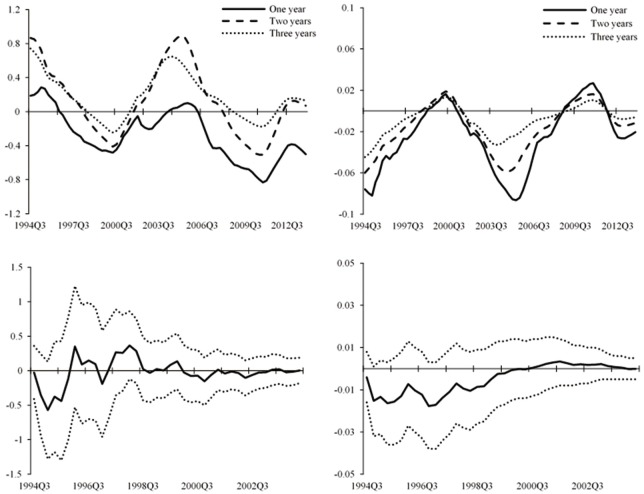
Impulse responses of asset prices to lending rate shocks in TVP-SVAR and simple VAR models. The figure shows impulse responses of stock market index (left) and the fundamental component of stock prices (right) to one *SD* innovation in the benchmark lending rate. The upper two figures are the results of TVP-SVAR model with three lines representing one-year (solid line), two-year (dashed line), and three-year (dotted line) horizon. The two figures below show the posterior mean (solid line) and 95 percent intervals (dotted line) for the simple VAR model.

Fig 8 displays the impulse responses of stock market index and the fundamental component to interest rate shocks for one- to three-year horizons over time in the TVP-SVAR model. We also present the impulse responses in the simple VAR to compare. We find that for the simple VAR model, the patterns of responses of stock prices to a tightening monetary policy have changed little over time and reduce to 0 around the year of 2004. In comparison, the impulse responses vary significantly over time in the case of TVP-SVAR model. The responses of the fundamental component to interest rate shocks are mainly negative, consistent with the consensus that the fundamental component decreases to a contractionary monetary policy. Nevertheless, the stock market index rises above its initial value in response to a monetary policy shock during the initial periods and stays positive for the majority of the sample periods. Although this result is not in accordance with what would be expected from the conventional view that asset prices will decrease following a monetary policy shock, it can be explained by the theory of rational bubbles discussed in Section “Method” According to this theory, the bubble component grows at the rate of interest rate. There are negative responses of the fundamental component and positive responses of the bubble component to the rise of interest rate, and the stock market index will also react positively when there is higher proportion of bubble component in the asset prices.

At the same time, we notice that movements of stock market index’s responses to monetary policy shocks are always in step with the trend of China’s stock market and the accumulation and bursting of the bubbles. Our sample period starts from the year of 1994, a period with high bubbles, when it displays the highest positive response of the stock market index to monetary policy shocks in the impulse response figure. This means that with high bubbles, monetary policy actions are not effective in controlling asset prices. In the following periods, bubbles burst and the figure shows that the impulse response goes down accordingly. After that, the China’s stock market embraces a rapid expansion following the reform of listed companies’ shareholder structure in 2006, when we can see in the figure that the response climbs up and leads a positive trend again.

Based on the above evidences, we can make the preliminary judgment that results we get from the impulse responses of stock market index to monetary policy actions indicate the existence of bubbles in asset prices and that monetary policy is not effective in controlling asset prices during periods of high bubbles.

**Fig 9:**
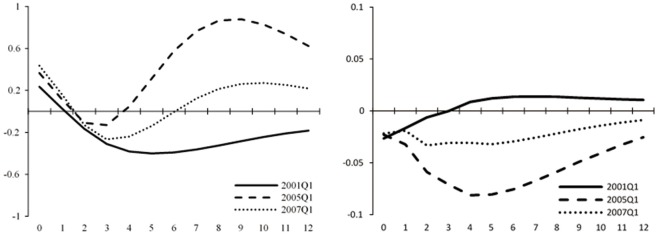
Impulse responses of asset prices to interest rate shocks at different points of time in the TVP-SVAR model. The figure shows impulse responses of stock market index (left) and the fundamental component of stock prices (right) to one *SD* innovation in the benchmark lending rate at different points of time. The black solid line shows the impulse responses of 2001*Q*1. The dashed line shows the impulse responses of 2005*Q*1. The dotted line is the impulse responses of 2007*Q*1.

Fig 9 illustrates the impulse responses at three different points of time, 2001Q1, 2005Q1 and 2007Q1 (shown by the black solid line, the dashed line, and the dotted line in the figures), which means that the initial time of the three lines are 2001Q1, 2005Q1 and 2007Q1, respectively. The lines show the time-varying impulse responses of asset prices to interest rate shocks in the following 4 years. Take the black solid line as an example, the initial period of t = 0 is 2001Q1, and the line shows the time-varying impulse responses of asset prices to interest rate shocks from 2001Q1 to 2004Q1 (t = 12). Similarly, the dashed line and the dotted line show impulse responses of stock index to interest rate shocks from 2005Q1 to 2008Q1, and 2007Q1 to 2010Q1. 2001Q1 and 2007Q1 are the high bubble periods, and 2005Q1 is a period following the long-term bear market and with less bubbles. These three points can in some degree represent the typical economic conditions in China’s stock market. Through these impulse responses, we can not only observe the effectiveness of monetary policy actions on asset prices at different points of time, but also validate the conclusions above by comparing different situations between high bubble periods and low bubble periods, if there is any.

We focus on results of the stock market index. For the case of 2001Q1, impulse response of the stock market index is positive in the initial period, indicating the presence of bubbles. Then it goes down and ends up negative, implying the contraction of bubbles. We can match this trend with the corresponding movement of the stock market’s 4-year-long downturn after the bursting of bubbles in 2001. Results of 2005Q1 also show a positive response of the stock market index to monetary policy shocks at first. After a short going down, there is a sharp rise, which is consistent with the accumulating bubble during the year 2006–2007, the largest bubble ever emerged in the history of China’s stock market. The high bubble during 2006–2007 can also be seen from results of 2007Q1. Compared to the previous two points, positive response of the stock market index to monetary policy shocks is significantly larger and more durable in 2007Q1. It turns negative after about three quarters, corresponding to the bursting of the bubble caused by the international financial crisis in late 2008. The negative response lasts for a year before it rises up again due to the gradual recovery of the stock market in China. We can see that the positive response in the following part of 2007Q1 is not that significant as in the case of 2005Q1. This can also be matched with the actual situation in China’s stock market that it has recovered during 2009–2010 but still hovers near 3000 points, lower than the highest 6000 points during 2006–2007.

**Fig 10:**
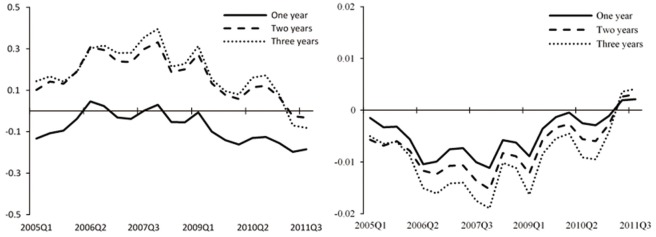
Impulse responses of asset prices to overnight interbank lending rate shocks in the TVP-SVAR model. The figure shows impulse responses of stock market index (left) and the fundamental component of stock prices (right) to one *SD* innovation in the overnight interbank lending rate for one-year (solid line), two-year (dashed line), and three-year (dotted line) horizons.

To test the applicability of the benchmark lending rate as the monetary policy indicator and the robustness of the above results, we use a more market-oriented monetary policy indicator—the overnight interbank lending rate—in the estimation of the model. The sample period is 2004Q2-2013Q4 due to the limitation of data availability. We get similar impulse response results displayed in Fig 10 that the positive responses of the stock market index rise with the accumulation of bubbles, and peak during 2007–2008, consistent with the actual stock market trend.

To conclude, there are evident bubbles in China’s stock market with time-varying scale and proportion. The TVP-SVAR model we apply can capture this character effectively, as can be seen from the impulse response functions that responses of the stock market index to monetary policy shocks fit the trend of China’s stock market and changes of the bubbles quite well. The empirical results show that influence of monetary policy actions on the stock market index depends on the comprehensive impact on the fundamental and the bubble component of asset prices, and monetary policy actions are not always effective in controlling the rising of asset prices during periods with high bubble proportion in China’s stock market.

## Empirical Results of Central Bank Communication

**Fig 11:**
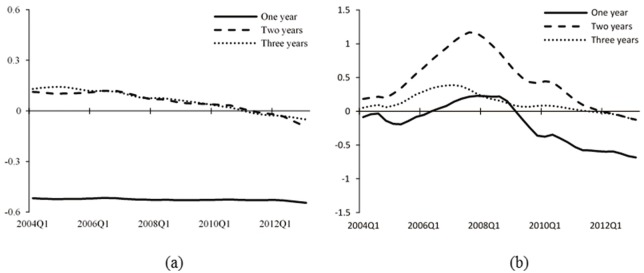
Impulse responses of asset prices to central bank communication and interest rate shocks. The figure shows impulse responses of stock market index to one *SD* innovation in the central bank communication indicator ([*a*]) and the benchmark lending rate ([*b*]) for one-year (solid line), two-year (dashed line), and three-year (dotted line) horizons. The sample period is 2003–2013.

**Fig 12:**
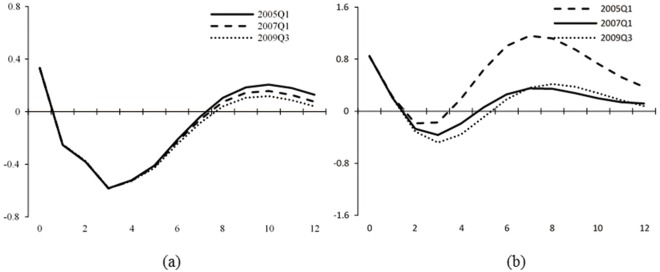
Impulse responses of asset prices to central bank communication and interest rate shocks at different points of time. The figure shows impulse responses of stock market index to one *SD* innovation in the central bank communication indicator ([*a*]) and the benchmark lending rate ([*b*]) at different points of time. The black solid line shows the impulse responses of 2005*Q*1. The dashed line shows the impulse responses of 2007*Q*1. The dotted line is the impulse responses of 2009*Q*3. The sample period is 2003–2013.

The empirical results of central bank communication are displayed in Figs 11 and 12. Fig 11 shows the impulse responses for one- to three-year horizons over time and Fig 12 displays the impulse responses at different points of time. It is worth mentioning that central bank communication has not been widely used by the PBOC until the year of 2003, since when the relative data is available. Therefore, the sample size is shorter than that of monetary policy actions. To make the results comparable, we show in Figs 11 and 12 that Panel [*a*] represents the results of central bank communication, and Panel [*b*] shows the results of benchmark lending rate for the same sample period of 2003–2013.

Fig 11 shows that both central bank communication and monetary policy actions have positive effects on the stock market index during the initial periods, indicating that effects of the two monetary policy instruments on asset prices with high bubble proportion are similar in the short term. However, when the following responses of stock market index to interest rate shocks keep climbing up with the accumulation of bubbles in the stock market after the year of 2003, the responses decline gradually in the case of central bank communication. This means that in the long run, during high bubble periods, even though monetary policy actions are not effective in controlling asset prices, central bank communication has more significant effects without causing drastic fluctuations in the stock market. Similar results appear in Fig 12, in which we focus on the impulse responses of 2005Q1, 2007Q1, and 2009Q3. Fig 12 also shows similar trends of the two monetary policy instruments during the initial period. However, during the subsequent periods, the negative responses of the stock market index are larger and more durable to central bank communication than those to monetary policy actions.

We consider the key reason is the effective guidance of investors’ expectation about the future monetary policy by central bank communication. Our results are in line with Gürkaynak et al. [21], who define central bank communication as the ‘future path’ factor of monetary policy and has relatively long-term effects on the economy. Sarno and Taylor [42] and Fratzscher [17] argue that central bank communication is able to reduce heterogeneity in the market’s information and expectations, and induce asset prices to move closely to the underlying fundamentals, which is called the ‘coordination channel’. Through this channel, central bank communication has longer-lasting effects because it changes the dynamics of the financial markets. In general, we suggest on the basis of the results here that central bank communication be used as an effective long-term instrument in the monetary policy toolkit.

In fact, panel [*b*] of Figs 11 and 12 is another robustness test for the conclusions we get in Section “Empirical Results of Monetary Policy Actions”. It applies a shorter sample period (2003–2013) for the estimation of asset prices and monetary policy actions and the impulse response results surely confirm the validity and accuracy of the results above.

## Concluding Remarks

In this paper, we compare the different effects of monetary policy actions and central bank communication on China’s stock price bubbles using a three-variable TVP-SVAR model with stochastic volatility. We get the following conclusions.

Monetary policy actions cannot effectively regulate the rising of stock prices during high bubble periods. With negative responses of the fundamental component and positive responses of the bubble component of asset prices to monetary policy shocks, the comprehensive impact of monetary policy depends on the relative proportion of the bubble. A contractionary monetary policy induces the stock market index to rise during periods of high bubbles.

Although central bank communication does not have significant short-term effects on asset prices either, it can restrain the excessive expansion of asset prices in the long run without causing drastic fluctuations in the stock market. In contrary to the effects of monetary policy actions, central bank communication is effective in controlling asset prices with high bubble proportion. According to our analysis, the key factor is its effective guidance to investors’ expectation about the future monetary policy. The central bank can make better use of communication as a long-term instrument.

## Supporting Information

S1 File Data used in the original manuscript. (XLSX)

S2 File Data used during the revision process. (XLS)
